# Multi-scale habitat assessment of pronghorn migration routes

**DOI:** 10.1371/journal.pone.0241042

**Published:** 2020-12-04

**Authors:** Andrew F. Jakes, Nicholas J. DeCesare, Paul F. Jones, C. Cormack Gates, Scott J. Story, Sarah K. Olimb, Kyran E. Kunkel, Mark Hebblewhite

**Affiliations:** 1 Faculty of Environmental Design, University of Calgary, Calgary, Alberta, Canada; 2 Montana Fish, Wildlife & Parks, Missoula, Montana, United States of America; 3 Alberta Conservation Association, Lethbridge, Alberta, Canada; 4 Montana Fish, Wildlife & Parks, Helena, Montana, United States of America; 5 World Wildlife Fund–Northern Great Plains, Bozeman, Montana, United States of America; 6 Wildlife Biology Program, W.A. Franke College of Forestry and Conservation, University of Montana, Missoula, Montana, United States of America; University of Sassari, ITALY

## Abstract

We studied the habitat selection of pronghorn (*Antilocapra americana*) during seasonal migration; an important period in an animal’s annual cycle associated with broad-scale movements. We further decompose our understanding of migration habitat itself as the product of both broad- and fine-scale behavioral decisions and take a multi-scale approach to assess pronghorn spring and fall migration across the transboundary Northern Sagebrush Steppe region. We used a hierarchical habitat selection framework to assess a suite of natural and anthropogenic features that have been shown to influence selection patterns of pronghorn at both broad (migratory neighborhood) and fine (migratory pathway) scales. We then combined single-scale predictions into a scale-integrated step selection function (ISSF) map to assess its effectiveness in predicting migration route habitat. During spring, pronghorn selected for native grasslands, areas of high forage productivity (NDVI), and avoided human activity (i.e., roads and oil and natural gas wells). During fall, pronghorn selected for native grasslands, larger streams and rivers, and avoided roads. We detected avoidance of paved roads, unpaved roads, and wells at broad spatial scales, but no response to these features at fine scales. In other words, migratory pronghorn responded more strongly to anthropogenic features when selecting a broad neighborhood through which to migrate than when selecting individual steps along their migratory pathway. Our results demonstrate that scales of migratory route selection are hierarchically nested within each other from broader (second-order) to finer scales (third-order). In addition, we found other variables during particular migratory periods (i.e., native grasslands in spring) were selected for across scales indicating their importance for pronghorn. The mapping of ungulate migration habitat is a topic of high conservation relevance. In some applications, corridors are mapped according to telemetry location data from a sample of animals, with the assumption that the sample adequately represents habitat for the entire population. Our use of multi-scale modelling to predict resource selection during migration shows promise and may offer another relevant alternative for use in future conservation planning and land management decisions where telemetry-based sampling is unavailable or incomplete.

## Introduction

Animals require habitat, defined as the abiotic and biotic resources and conditions that promote occupancy and population persistence, to meet annual life-history requirements [1]. Study of wildlife-habitat relationships has brought forth the importance of spatial scale as a nested hierarchy of lenses through which to view animal preferences [2]. At broad spatial scales, the boundaries of species’ ranges convey one scale-specific definition of habitat while at fine scales the use of individual food items convey another. Definitions of habitat also include a component of temporal scale, from broad seasonal requirements to diurnal cycles of behavior-mediated habitat needs [3]. Intertwined within all of these spatiotemporal definitions of habitat are the movements of animals themselves, or the means by which they access such resources. Animal movements also range in scale from individual bites of twigs on a plant to intercontinental migrations between seasonal ranges. Here, we study the habitat selection of animals during seasonal migration; a specific and important period in an animal’s annual cycle associated with broad-scale movements. However, we further decompose our understanding of migration habitat itself as the product of both broad- and fine-scale behavioral decisions.

Seasonal migration (hereafter “migration”) has been defined as repeated annual movement typically during spring and fall, which connects discrete geographic areas of habitat used at different times of the year [4]. Similar to resource selection while on seasonal range, migration is also a multi-scale process, with resource selection occurring across multiple spatiotemporal scales [5,6]. We define a “migration route” as the integration of migratory habitat selection patterns of fine-scale (third-order) with those at the broader scale (second-order). However, resource selection may differ between seasonal and migration periods because migration routes tend to be linear in shape and occur over a much shorter time period (i.e., weeks compared to months on seasonal range). Consequently, migration routes can be a useful focus of conservation efforts geared towards maintaining connectivity and population persistence. In some applications, migration routes are mapped according to the relative densities of telemetry location data from a sample of animals, with the assumption that the sample adequately represents habitat for the entire population [7]. However, this approach may become problematic in situations when sampling of populations is incomplete or unevenly distributed, misrepresenting population-level importance in predictions. Multi-scale habitat modelling to study and predict migration routes may offer another relevant alternative for use in future conservation planning and land management decisions where telemetry-based sampling is unavailable, incomplete or uneven.

Recent analytical approaches such as step selection functions (SSF) are aimed to predict movement-based habitat selection at the fine scale of individual movement steps [8]. Within a used-available SSF design [8] ‘used’ locations are compared to putative ‘available’ locations within a spatiotemporal window defined in scale by measurements of each individual step. To design applicable multi-scale movement studies, researchers must define availability accordingly at different spatiotemporal resolutions (e.g., a hierarchical nested approach) to understand how selection changes across scales [9–11]. Once such hierarchically nested multi-scale models are produced, integrating these into a single scale-integrated prediction (i.e., a map) for conservation and management is then possible [10,12]. Multi-scale models have been found to more accurately predict habitat selection than single scale models for species [13], and for example, have been integrated into spatial depictions of habitat for woodland caribou (*Rangifer tarandus caribou*) seasonal range selection [10]. Despite advances in multi-scale resource selection models and recent recommendations [14], few have been integrated across scales with specific focus on the study of movement [15–17].

Pronghorn (*Antilocapra americana*) are an indigenous ungulate that ranges across the globally threatened grassland and shrub steppe communities of western North America [18,19]. These communities face cumulative ecological threats from anthropogenic development, which may disrupt natural ecological processes (i.e., animal daily and seasonal movements, connectivity) and result in diminished wildlife populations [4,20,21]. Pronghorn populations are often partially migratory where some portion of the population migrates, and others remain year-round residents [22–24]. Aside from barren-ground caribou (*Rangifer tarandus groenlandicus*) and mule deer (*Odocoileus hemionus*), pronghorn have exhibited the longest migrations among North American ungulates [24], and are particularly sensitive to human-caused habitat fragmentation. For example, 75% of pronghorn migrations into the Greater Yellowstone Area have been lost over the last decades from cumulative anthropogenic factors [4]. Furthermore, a fine-scale study of extant pronghorn migration pathways has indicated the potential for local avoidance of anthropogenic features such as housing developments and roads [25].

In addition to their practical application for conservation efforts, multi-scale assessments of habitat selection can also reveal which factors are most limiting to species dynamics [10]. Following this logic, features affecting selection at broad scales have stronger impacts on dynamics, whereas those affecting fine-scale selection have relatively less impact [26]. Alternatively, factors consistently selected across multiple scales could indicate strong importance to a species. In the case of pronghorn, both natural and anthropogenic features have been shown to influence habitat selection at different spatial scales. In studies of selection within home ranges, researchers showed pronghorn avoided human development at large spatial scales [27], while others showed variable responses of pronghorn to human development at finer spatial scales [28,29]. Anthropogenic features may also influence selection during migration with potential to cause shifting or cessation altogether of pronghorn seasonal migration in some landscapes [30]. Here we use a multi-scale assessment of pronghorn migration habitat to compare factors affecting migration routes at broad versus fine scales, with specific attention to impacts of a variety of anthropogenic features.

We examine multi-scale migration-specific habitat selection of pronghorn between discrete summer and winter ranges at the northern periphery of their range in the Northern Sagebrush Steppe (NSS) of North America. Our objectives were to develop multi-scale resource selection models during both spring and fall migrations, relative to environmental gradients and anthropogenic factors. First, we assessed resource selection at two spatial scales; the fine-scale selection of sequential steps along the pathway (third-order) and then at the broad-scale of a migratory neighborhood encompassing the pathway and its surroundings (second-order). We then integrated and mapped predictions across these two spatial scales, representing the migration route, and validated how well a single scale-integrated spatial map could predict fine-scale use within a broad-scale landscape. During both migrations periods we predicted that pronghorn would follow spatiotemporal gradients in forage productivity (phenology) [31,32] along steps within the migratory pathway (third-order), while at the scale of a migratory neighborhood (second-order), we predicted pronghorn would select for native grasslands in less rugged areas to maintain energy reserves required for migration [25,33]. We also predicted pronghorn would avoid higher densities of roads and oil and natural gas wells during migration [28,34,35]. Lastly, we predicted seasonal variation with a stronger effect of forage phenology during spring green-up compared to fall migration [24,36].

## Material and methods

### Study area

**Fig 1:**
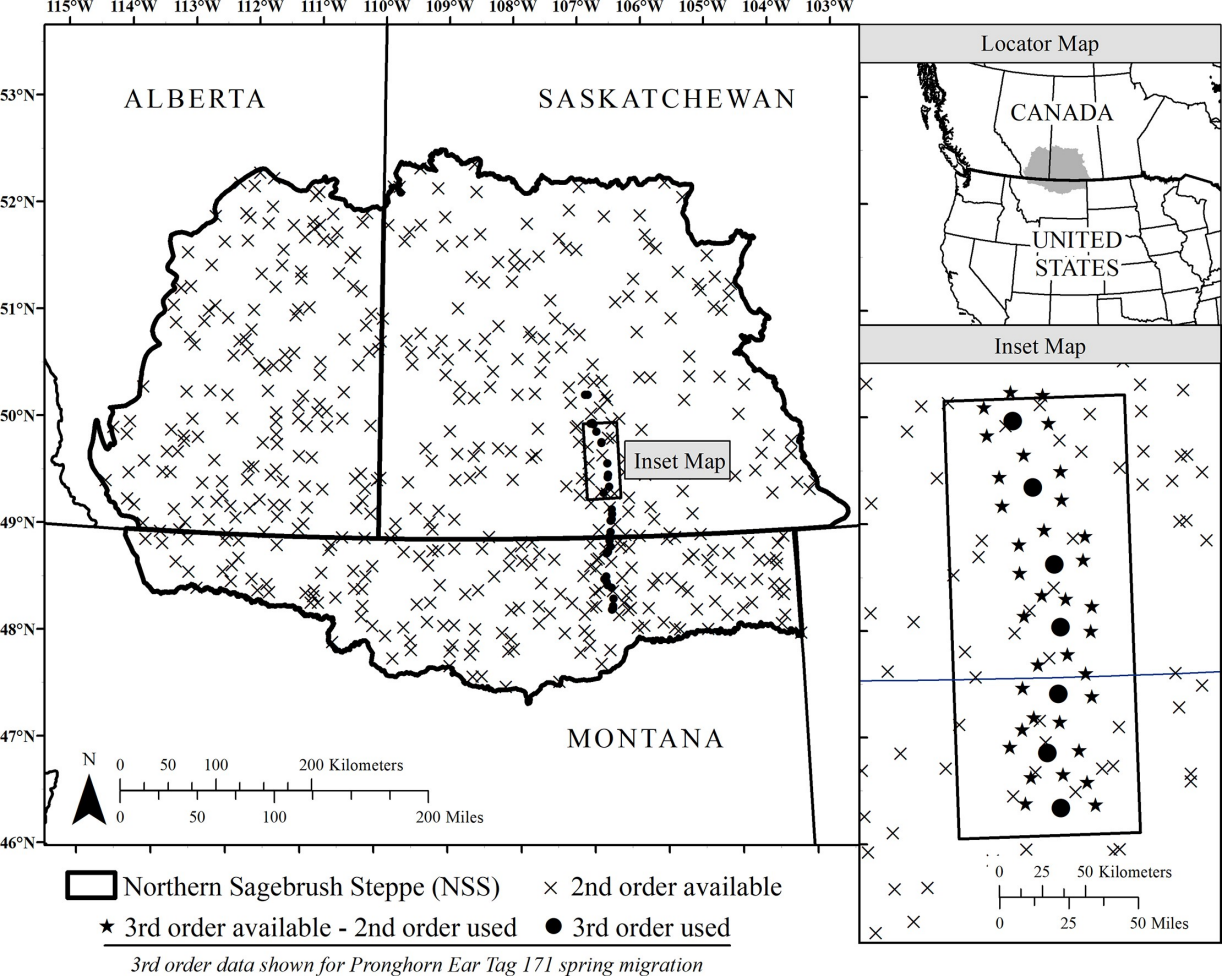
An example of the hierarchically nested used-available design for one individual (Pronghorn Ear Tag 171) to predict pronghorn (*Antilocapra americana*) migratory route habitat selection during spring at two scales across the Northern Sagebrush Steppe. Pronghorn second-order selection compares migratory neighborhoods to the entire study area whereas third-order selection compares migratory pathway relocations to the migratory neighborhood. Scales of migration habitat selection are nested as a result of third-order available locations also operating as used locations at the broader, second-order scale. This original map was created using ArcGIS Desktop version 10.4.1 (ESRI, Inc., Redlands, California, USA) and includes vector data from Natural Earth (“Admin 1 –States, Provinces”) which complies with CC BY 4.0 license; available from https://protect-us.mimecast.com/s/pxvaCxkVBDILmvE2uAfAL2?domain=naturalearthdata.com.

We studied pronghorn migratory habitat selection across the Northern Sagebrush Steppe during 2003–2011. The study area encompassed 315,876 km^2^ of the prairie regions of Alberta, Saskatchewan, Canada and Montana, USA (Fig 1). The landscape is flat with open plains and rolling hills where rivers and other waterways have exposed badlands, creating deep coulees throughout the region [37]. Vegetation types across the region include a mosaic of native prairie, tame pastures, and irrigated and dryland agricultural fields. Land administration and development vary with a mix of 50:50 private to public land ownership, and a mix of land use throughout the region. See [24,38] for further details.

### Pronghorn capture and migratory pathway identification

In this subsection, we identify protocols, equipment and settings, as well as analytical approaches to parcel relevant pronghorn movement data for subsequent analysis. We used helicopter net-gunning to capture 185 female pronghorn during the winters of 2003–2010 [39] and deployed global positioning system (GPS) collars programmed to collect locations every 2 (*n* = 64) or 4 (*n* = 121) hours (Lotek GPS 3300 and ARGOS 7000SA models; Lotek Wireless, Newmarket, Ontario, Canada). Of the 185 individuals captured, data were successfully retrieved from 173 individuals. Habitat-induced GPS bias was negligible in our study as collars obtained a 98% fix-success rate [40]. Details of capture and handling were described previously [24,38] and followed protocols approved by the Alberta Wildlife Animal Care Committee (#11861, #16707, #20394), Montana Fish, Wildlife & Parks Animal Care and Use Committee (#11–2007), and the Saskatchewan Ministry of Environment (#09FW040).

We first classified individual pronghorn’s migratory pathways using non-linear models of net squared displacement (NSD), which measures the straight-line distance between the starting and each successive location along an individual’s movement pathway [24,41]. We used NSD data and graphical outputs to classify individuals as migratory, mixed-migratory, or resident using daily locations for each pronghorn. For the purposes of this paper, individuals classified as mixed- migratory were considered migratory, while individuals classified as residents were excluded from subsequent analyses. We identified start and end dates of migration (both spring and fall) based upon variation in NSD from locations on winter range [24]. Individual migratory pathways with < 20 observations (*n* = 23) were not considered for further analysis due to insufficient data [42]. Stopover sites were distinguished from migratory pathways based on step lengths along the overall pathway and we included only a single location at each stopover site, removing excess repeated GPS location at each site from the analysis [24]. We removed repeated locations collected within stopover areas from the migratory pathway as we were interested in only the selection patterns made during migration (i.e., distinctly moving along a trajectory) and not while temporarily sedentary during stopovers. We pooled data across years for a small subset of individuals (*n* = 7) for which two years of data during a particular season were collected.

Of the 173 individuals for which we were able to retrieve collars, we used data from 170 animal-years, defined as an individual’s annual movement. Of the 170 animal-years, 94 (55%) exhibited seasonal migrations (either migratory or mixed-migratory) and 76 (45%) were classified as resident. The 94 animal-years that exhibited seasonal migration, undertook spring migrations with mean start and end dates of March 22 and April 10 respectively, lasting on average 20 days [24]. Of the 94 animal-years that exhibited seasonal migration, 70 fall migrations were completed, with mean start and end dates of October 31 and November 10 respectively, lasting on average 11 days [24].

### Sampling framework and analysis

In this subsection, we conceptualize the sampling framework, specify covariates and our approach to modelling pronghorn migratory route habitat. We adapted a previous hierarchical habitat selection framework to assess multi-scale selection of migration pathways by pronghorn [13]. We defined migratory pathways as the subset of GPS locations between the start and end date of each individual’s seasonal migration and excluded repeating locations identified as local movements within stopover sites [24]. We next defined a broader scale “migratory neighborhood” as the area surrounding each migratory pathway and within a single step-length of all locations. Using these migratory pathways and neighborhoods, we assessed pronghorn selection of migration habitat at two spatial scales (Fig 1); broad-scale selection of migratory neighborhoods relative to the entire study area (second-order) and fine-scale selection of local sites along the migratory pathway (third-order) relative to the surrounding neighborhoods. At the finer third-order scale, we used a movement-based approach based on step selection functions (SSF) [43], a type of resource selection function (RSF) [44], whereby used locations were compared to matched available locations in immediate proximity to each used location. We paired used migratory pathway locations for each pronghorn to a matched sample of available locations drawn at a 5:1 ratio to characterize the surrounding migratory neighborhood. We used individual empirical step length and movement angle distributions from each pronghorn to generate movement-defined matched available locations for each pronghorn used location [45]. Matched, randomly drawn locations were weighted with a value of 0.2 per location to retain 1:1 sample sizes of used and available samples in subsequent analyses (Fig 1). To nest this third-order model within a broader second-order model of habitat selection, we then pooled each individual’s migratory neighborhood sample of available locations together and treated them as individual-level samples of use at the second-order scale for comparison to the entire study area, *sensu* [10]. That is, at this broader scale, we compared used locations within the pooled set of migratory neighborhoods (i.e., available third-order locations) to an unmatched set of available points drawn from within the entire study area at a 1:1 ratio (Fig 1).

We used a suite of environmental and anthropogenic variables to characterize spatial variation across the Northern Sagebrush Steppe with potential relevance to migrating pronghorn. We integrated spatial data from Canada and the U.S., which included a suite of layers characterizing land cover, topography, hydrology, anthropogenic features, and vegetation productivity. Specifically, we used a digital elevation model (DEM; 30 m resolution) to derive aspect, slope, and a vector ruggedness measure characterizing terrain ruggedness based on variability in aspect and the gradient component of slope (VRM) [46] for each point. We characterized land cover type (30 m resolution) into 10 categories (annual cropland, conifer forest, deciduous forest, developed, exposed, grassland, pasture and perennial cropland, shrubland, water, and wetland), with annual cropland identified as the reference variable, by combining data from Canada (Agriculture and Agri-Food Canada) and Montana (Montana Spatial Data Infrastructure (MSDI) Land Use/Land Cover), and determined which land cover category each point fell within. We used Moderate Resolution Infrared Spectroscopy (MODIS) 16-day composite normalized difference vegetation index (NDVI) images (250 m resolution) to measure vegetation phenology [47,48] and MODIS 8-day composite snow extent images (500 m resolution) to measure snow cover [49,50]. MODIS data were mosaicked and pre-processed using cloud-correction algorithms [51]. We created yearly 16-day tiles (*n* = 272) for NDVI and yearly 8-day tiles (*n* = 544) for snow cover, and estimated NDVI and snow cover for each pronghorn used and available location using NDVI and snow cover tiles specific to the time during which locations were collected. We measured stream density (m per m^2^) at each location using neighborhood analysis of vector hydrology feature data mapped at the 1:1,000,000 scale. We similarly quantified densities (m per m^2^) of paved and unpaved roads using agency data compiled from Alberta Sustainable Resource Development, Saskatchewan Ministry of Environment, and the Montana Department of Transportation. We likewise quantified densities (#wells per m^2^) of oil and natural gas wells from data provided by Alberta Sustainable Resource Development, Saskatchewan Energy and Resources, and the Montana Board of Oil and Gas. We accounted for temporal variation in wells being drilled relative to conditions present when each pronghorn GPS location was collected. First, we calculated the well densities specific to time period across the study period and second, calculated the distance of each used and available location from wells actively being drilled within a temporal window of 16 days to account for human activity during drilling and extraction [52]. We also evaluated quadratic terms for road and slope variables to account for possible non-linear responses by pronghorn [28,35].

At the second-order we used generalized linear mixed-effect models (GLMMs) with a random intercept for each individual to model pronghorn migratory neighborhood selection [53]. We fit GLMMs with the logit link comparing pooled individual data for each seasonal migratory neighborhood (spring, fall) to a set of random locations drawn from across the study area. For third-order modeling of local sites along each individual’s migratory pathway, we used conditional fixed-effect logistic regression to account for the matched design in this case, specifically comparing the attributes used at each migratory step to those immediately surrounding [54]. We centered and standardized variables prior to modelling for each order of selection and season [55]. Prior to each analysis, we screened variables univariately by comparing each variable separately to the null model and included all variables with moderate statistical significance (*P* < 0.20) in subsequent multi-variable analyses [42]. We then screened the remaining candidate variables for multi-collinearity using a correlation coefficient threshold of |*r*| ≥ 0.7 and removed one of the paired correlated variables from further analysis. We then used manual backwards stepwise logistic regression to individually withdraw insignificant variables (*P* > 0.05) until models were reduced to include only significant variables to obtain final global model estimates [56].

### Multi-scale model validation and spatial predictions

In this subsection, we describe model validation procedures for mapping single and multi-scale predictions. We evaluated the performance of second- and third-order top models in predicting pronghorn seasonal migrations using *k*-fold cross validation [57]. We iteratively withheld 20% of individuals and re-fit final models with the remaining 80% of individuals across each of 5-folds of data. For each fold, we estimated the Spearman rank correlation between the predicted relative probabilities of occurrence in 10 ordinal habitat categories, and the observed frequency of testing data within each category [57].

We created spatial predictions of the population level second-order model across the Northern Sagebrush Steppe [12]. Next, we created spatial predictions of the population-level third-order model, estimating the relative probability of local site selection for migratory pathways, pending occurrence within a greater migratory neighborhood [8]. When making spatial predictions at both scales, we limited the maximum and minimum values of each covariate across the NSS to be no less or greater than the minimum and maximum values sampled within our analyzed data. Fixing the minimum and maximum covariate values constrains model predictions to remain within the range of conditions sampled by our analyses [10]. When making model predictions, we set time-dependent variables to central values for the spring and fall periods (e.g., NDVI tiles: spring, March 22 –April 6; fall, November 1 –November 16) [24]. We assumed that no wells were actively being drilled for the predicted spatial maps and thus predictions did not account for the distance to wells actively being drilled. Lastly, we used a linear stretch to rescale relative predicted probabilities for each predicted raster between 0 and 1 [10,12].

We multiplied the unstretched second- and third-order scaled maps to create a scale-integrated step selection function (ISSF) spatial map output and then used linear stretch to rescale ISSF predicted values between 0 and 1 [10,12]. For example, if a pixel had a relative probability of selection value at the second-order of 0.80 and the same pixel had a relative probability value at the third-order of 0.50, then the combined ISSF relative probability value would be 0.40. Lastly, we again used *k*-fold cross validation to specifically validate the predictions of the ISSF. For this test, we assessed the ability of the model to integrate across scales and predict fine-scale use (migration pathways) relative to the broad-scale landscape (study area), and thus assessed the alignment of withheld GPS locations to ISSF predictions made across the entirety of the study area. Spatial and statistical analyses were conducted using a combination of ArcGIS 10.1 (ESRI 2012), the Geospatial Modelling Environment (GME; Beyer 2013), and Program R (v. 3.5.1; R Core Team 2013)) with the lme4 (v. 1.1–19) and survival (v. 2.43) packages [58,59].

## Results

### Second-order selection of migratory neighborhoods

**Fig 2:**
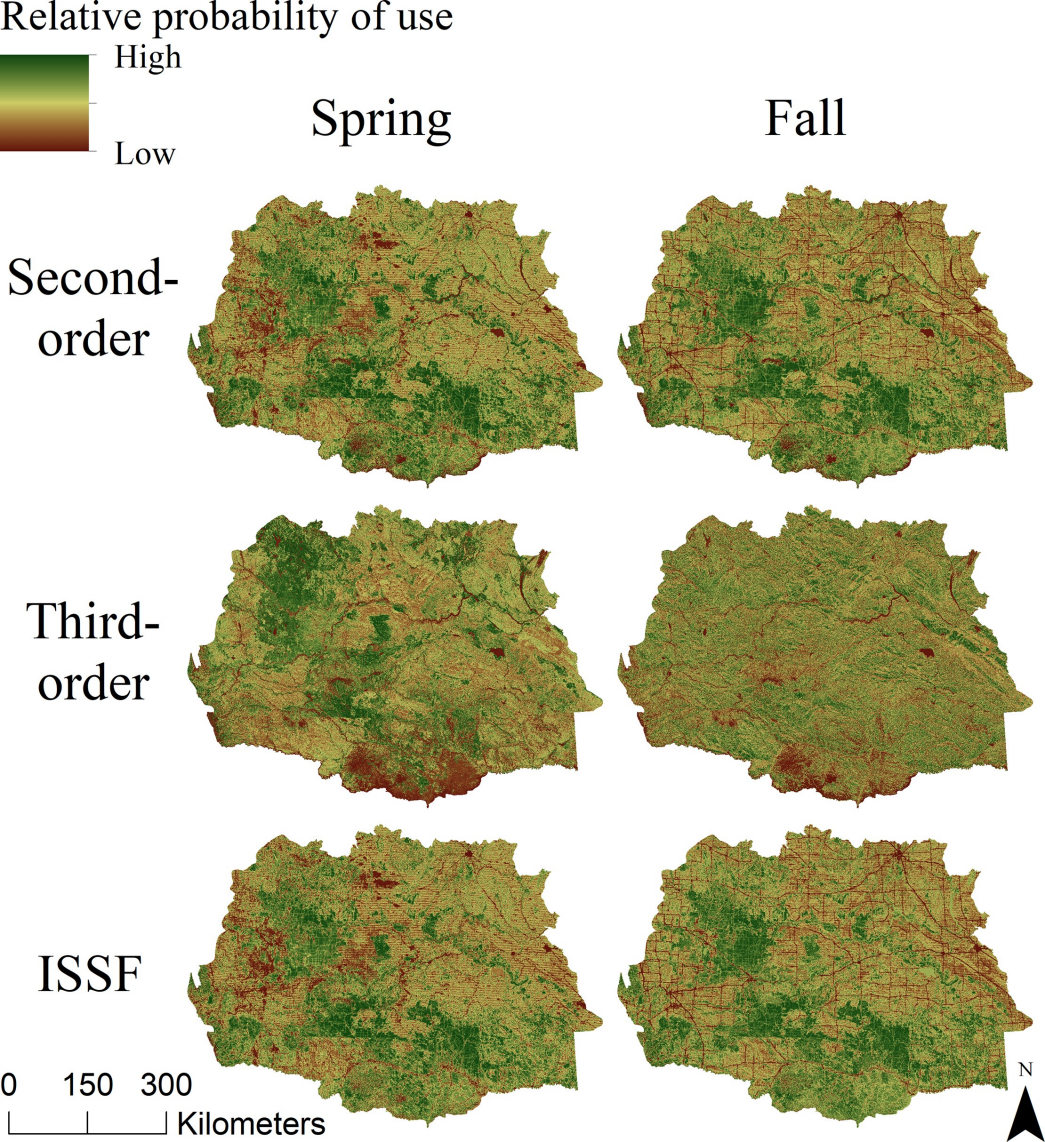
Resource selection spatial predictions for female pronghorn across the Northern Sagebrush Steppe from 2004–2010 during spring and fall migrations. Second-order selection during spring and fall are identified in the first row, third-order selection during seasonal migrations are identified in the second row, and scale-integrated step selection function (ISSF) map during seasonal migrations are identified in the third row. ISSF was calculated by multiplying the second- and third-order selection values across the Northern Sagebrush Steppe into a scale-integrated spatial prediction. This figure presents original map predictions that predicts the probability of use by pronghorn where green pixels indicate high probability areas for migration habitat, while red pixels indicate low probability areas.

**Fig 3:**
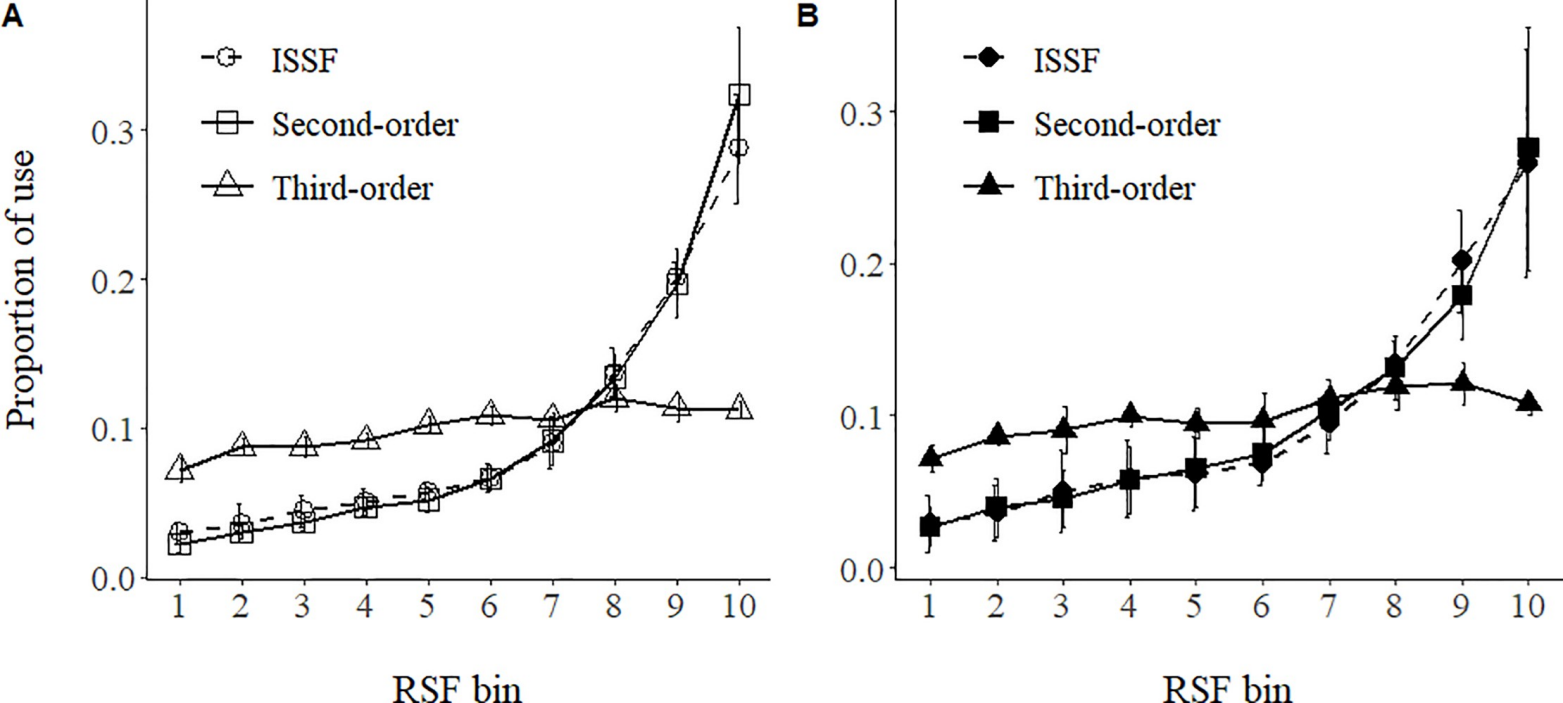
Pronghorn predicted relative probabilities of occurrence within ten ordinal habitat categories binned by resource selection probability functions during spring (A) and fall (B) for second-, third-, and scale-integrated step selection function (ISSF) spatial maps. Using *k*-fold cross validation, maps indicate that second-order models and ISSF scale maps during both seasons were more effective in discriminating between low versus high quality (bins 8–10) migration habitat than third-order models.

Second-order scale models generally indicated broad-scale selection for or avoidance of a number of environmental and anthropogenic variables (Table 1). Among land cover types, grassland was the most preferred and conifer forest was the most avoided during both spring and fall seasons Table 1. Standardized model coefficients suggested that selection for increased vegetative greenness (i.e., NDVI) was among the strongest drivers of broad-scale migration habitat for pronghorn during spring migration, but not selected during fall. Pronghorn also generally selected for southern aspects and avoided steep or rugged slopes during both seasons S1 Fig, while appearing to avoid large hydrologic features during spring but select for them during fall. Interestingly, snow cover was not retained in either the spring or fall models. With regards to human disturbance, pronghorn avoided paved roads during both seasons, avoided unpaved roads during spring, and below densities of 0.0015 m/m^2^ during fall, respectively, and avoided those areas of high well density and in close proximity to active wells only during spring Table 1; S1 Fig. Spring and fall second-order predictive maps identified large areas of native grasslands and sagebrush within a mosaic of land use as prioritized migratory neighborhoods (Fig 2). Evaluation of the predictive capacity of second-order models revealed excellent discrimination of the relative ranking of habitats across the study area during both seasons (ρ = 1.00, *P* <0.001; Table 2; Fig 3).

**Table 1:** Top second-order resource selection function model standardized coefficients for female pronghorn migratory neighborhood selection during spring and fall migration across the Northern Sagebrush Steppe, 2004–2010.

	Spring Migration				Fall Migration			
Variable	β	SE	Z	*P*	β	SE	Z	*P*
Intercept	-0.350	0.026	-13.339	<0.001	-0.384	0.040	-9.582	<0.001
Landcover type^a^								
Water	-1.009	0.183	-5.521	<0.001	-1.662	0.320	-5.190	<0.001
Exposed	0.897	0.118	7.609	<0.001	0.575	0.174	3.298	0.001
Developed	0.394	0.202	1.953	0.051	0.447	0.322	1.388	0.165
Shrubland	-0.226	0.075	-2.998	0.003	0.056	0.113	0.501	0.617
Wetland	-0.037	0.123	-0.303	0.762	-0.101	0.194	-0.518	0.604
Grassland	0.967	0.034	28.116	<0.001	0.932	0.053	17.747	<0.001
Pasture and perennial crop	-0.341	0.053	-6.399	<0.001	0.002	0.077	0.030	0.976
Conifer	-2.567	0.568	-4.518	<0.001	-2.906	1.146	-2.535	0.011
Deciduous	-1.138	0.227	-5.018	<0.001	-0.703	0.323	-2.176	0.030
NDVI	0.240	0.016	15.437	<0.001	-	-	-	-
Topographic variation								
VRM^b^	-0.095	0.022	-4.422	<0.001	-	-	-	-
Slope	0.219	0.027	8.127	<0.001	0.253	0.041	6.221	<0.001
Slope^2^	-0.088	0.008	-10.483	<0.001	-0.090	0.013	-7.197	<0.001
Aspect	-0.072	0.014	-5.198	<0.001	-0.193	0.022	-8.788	<0.001
Hydrologic density	-0.109	0.014	-7.552	<0.001	0.051	0.023	2.284	0.022
Anthropogenic features								
Paved road density	-0.162	0.024	-6.780	<0.001	-0.317	0.039	-8.181	<0.001
Unpaved road density	-0.403	0.017	-23.711	<0.001	-0.293	0.027	-10.911	<0.001
Unpaved road density^2^	0.044	0.009	4.820	<0.001	0.086	0.015	5.721	<0.001
Well density	-0.084	0.015	-5.435	<0.001	-	-	-	-
Distance to active drilling of wells	0.133	0.014	9.187	<0.001	-	-	-	-

^a^Annual crop is the reference category.

^b^VRM = Vector Ruggedness Measure.

**Table 2:** Spearman rank-based model validation results for second-order, third-order, and scale-integrated step selection function (ISSF) models of female pronghorn migrations across the Northern Sagebrush Steppe, 2004–2010.

	Spring		Fall	
	Spearman ρ	*P*	Spearman ρ	*P*
second-order	1.00	<0.001	1.00	<0.001
third-order	0.939	<0.001	0.879	0.001
ISSF	1.00	<0.001	1.00	<0.001

ISSF was calculated by multiplying the second- and third-order selection values across the Northern Sagebrush Steppe into a scale-integrated spatial prediction. Results are based on *k*-fold cross-validation of top models for each season and scale using *k* = 5 folds to iteratively refit best models and validate using withheld data.

### Third-order selection of migratory pathways

Selection of migration pathways at the finer, third-order scale indicated weaker and less consistent patterns across the spring and fall seasons in comparison to second-order selection. Relative to the reference category annual cropland, pronghorn selected for grassland and avoided water, wetlands, and deciduous forest during spring. However, during fall, only the avoidance of water and developed areas were significant relative to annual croplands. Similar to patterns in second-order models, pronghorn selected for increased vegetative greenness (NDVI) at fine scales in the spring but did not select this covariate in the fall. Pronghorn selected for southern aspects and avoided rugged terrain (VRM) at the third-order across both seasons Table 3; S2 Fig. As in second-order models, snow cover was not retained in final migratory pathway models. Contrary to patterns of selection at the broader second-order, pronghorn showed no significant responses during either migration period to any measures of human disturbances, whether roads or wells, at the third-order scale. Spring and fall third-order predictive maps identified migratory pathway habitat of both high and low relative probability of use with generally more northerly areas across the Northern Sagebrush Steppe identified as prioritized habitat during spring and more southerly areas identified as lower quality habitat during fall (Fig 2). Evaluation of model predictions showed a strong correlation between relative model predictions and the proportion of withheld telemetry locations during *k*-fold cross-validation during both spring (ρ = 0.939, *P* <0.001) and fall (ρ = 0.879, *P* = 0.001; Table 2). However, visual inspection of the relationship between model predictions and the frequency of withheld data suggest that the discrimination across 10 bins of predicted habitat quality was relatively weaker for third-order models in comparison to second-order models. Overall, we interpret this to indicate that predictions of third-order models did indeed correlate to increased habitat quality but model validation suggests that realized differences in quality among predicted bins were less certain and/or lower in magnitude than was the case for second-order models (Fig 3).

**Table 3:** Top third-order step selection function conditional logistic regression model coefficients for pronghorn migratory pathway selection during spring and fall migration across the Northern Sagebrush Steppe, 2004–2010.

	Spring Migration				Fall Migration			
Variable	β	SE	*Z*	*P*	β	SE	*Z*	*P*
Landcover type^a^								
Water	-0.665	0.260	-2.552	0.011	-2.037	1.004	-2.029	0.042
Exposed	-0.039	0.078	-0.497	0.619	0.094	0.116	0.809	0.419
Developed	-0.074	0.159	-0.469	0.639	-0.806	0.394	-2.046	0.041
Shrubland	-0.075	0.061	-1.239	0.215	-0.070	0.099	-0.708	0.479
Wetland	-0.428	0.124	-3.462	0.001	-0.259	0.187	-1.388	0.165
Grassland	0.049	0.024	2.091	0.037	-0.005	0.038	-0.132	0.895
Pasture and perennial crop	0.060	0.042	1.440	0.150	-0.051	0.069	-0.741	0.459
Conifer	-0.360	0.712	-0.506	0.613	-12.021	578.985	-0.021	0.983
Deciduous	-1.023	0.380	-2.691	0.007	0.014	0.285	0.049	0.961
NDVI	0.042	0.010	4.268	<0.001	-	-	-	-
Topographic variation								
Aspect	-0.018	0.009	-1.899	0.058	-0.078	0.016	-4.787	<0.001
VRM^b^	-0.104	0.015	-7.088	<0.001	-0.110	0.024	-4.629	<0.001

^a^Annual crop is the reference category.

^b^VRM = Vector Ruggedness Measure.

### Scale-integrated predictions of migration route habitat

In general, scale-integrated predictions of pronghorn migration route habitat (i.e., ISSF maps) displayed similar core areas to one another during spring and fall migratory periods across the Northern Sagebrush Steppe (Fig 2). Predicted maps for both seasons highlighted the importance of large grassland areas as migration route habitat as well as the avoidance of human disturbance regimes. Validation of the ISSF maps showed excellent ability of this approach to rank migration route habitat across the entire study area with relevance to fine-scale use by pronghorn, as measured by concentration of telemetry data and that pronghorn migratory route predictions were mainly governed by second-order selection (Table 2; Fig 3).

## Discussion

Wildlife exhibit varying resource requirements dependent on the scale of selection and season [2]; accordingly, it is important to not only assess seasonal range selection (i.e., summer or winter) but to identify significant factors that affect migratory selection patterns at varying scales. Selection at broader scales may indicate those features most limiting to species distributions or population dynamics [26]. Furthermore, similar selection patterns across scales may reveal the importance of those features to the species. In our study, we applied scale-integrated resource selection mapping methods to merge pronghorn migratory requirements across scales. Combining migration habitat predictions across scales has been recommend in habitat selection literature [14], yet rarely achieved [17]. We nested our predictions of habitat relationships along finer scale migratory pathways within a broader scale context of migratory neighborhoods to more functionally depict migratory route habitat. To our knowledge, this approach has not been used for other migratory ungulates in open prairie landscapes.

We found strong avoidance of human disturbance features during both migratory periods at broad spatial scales of migratory neighborhoods, but once on a migratory pathway, pronghorn were not strongly or consistently affected by human disturbance. In addition, we found selection for grasslands across scales during spring and at broader scales only during fall migratory periods, but surprisingly not NDVI, which was only included in spring migration models. Our results vary to some degree to those reported by [35] for our migratory pronghorn during summer and winter seasons. They found migratory pronghorn in the Northern Sagebrush Steppe to consistently select against paved and all roads across both seasons and scales of selection, while roads where only important at the broad-scale during our study [35]. During summer, NDVI was significant across scales for migratory pronghorn, which is only consistent with our spring migration period results [35]. These differences in selection patterns on seasonal ranges versus migratory routes for the same individuals affirms the need to assess and manage migratory routes separately from seasonal range selection.

Disturbance features, including road, oil, and natural gas well densities, were a significant factor in determining pronghorn migration habitat across the Northern Sagebrush Steppe. The pronghorn’s capacity to constantly and rapidly move allows individuals to minimize exposure, specifically in response to oil and natural gas extraction, to less than ideal conditions during migration if resources are available. Within a relatively short period, pronghorn may transverse areas to access more favorable conditions (i.e., areas near wells). Nonetheless, roads, oil, and natural gas wells are anthropogenic features with both direct and indirect effects on wildlife and ecosystem processes [20,60]. Other studies have shown anthropogenic features can alter ungulate behavior and habitat suitability [28, 61,62]. Our results showing avoidance of roads during both migratory seasons are consistent with others that suggest roads, and the accompanying high-vehicle densities and speeds on primary and secondary roads, may act to fragment the landscape more than any other measured factor [28,34]. Roads, well development, and associated vehicle traffic can reduce habitat suitability resulting in avoidance and increased vigilance during migration. These factors can affect the direction, distance, and timing of pronghorn migration [28,63]. Continued anthropogenic development may incrementally degrade current migration habitat and cause pronounced alterations in available habitat needed to sustain long-distance movements. Further research is needed to elucidate the separate influences of roads and other linear infrastructure such as fences, railways and canals on pronghorn behavior during migration, and designing approaches for mitigating the effects of these features.

Grasslands and NDVI were selected for at both scales only during the spring migratory period, but their relative importance switched depending on scale of selection. At the broad-scale (second-order) pronghorn showed stronger relative selection for grasslands while at the finer scale (third-order) pronghorn showed stronger relative selection for NDVI. That is, grasslands influenced the selection of the migratory neighborhood, but once on a migratory path the “greenness” of the path (as measured by NDVI) affected pronghorn selection. Grasslands provide foraging opportunities during spring, but importantly also provide large, un-fragmented tracts to migrate through that are relatively undisturbed by humans [64,65]. As with other ungulates, during spring migration female pronghorn seek and consume forage containing peak protein levels, which provide critical nutrition to replenish tissue reserves depleted during the preceding winter [66,67]. Ungulates have been observed to follow seasonal “green-waves” of plant phenology along elevation or latitude gradients [36,68]. At the landscape scale, greenness may be patchily distributed, influenced by soil, moisture, and terrain features, such as aspect and slope. Using NDVI as a measure to represent the forage quality of newly sprouted grasses and forbs, research has shown that grasslands had higher productivity than crop lands [69]. Tracking forage quality is an important aspect of ungulate migration patterns [36,66,70] and we found it played a significant influence on pronghorn migration habitat selection in the Northern Sagebrush Steppe at multiple scales during the spring. There was a definite latitudinal and temporal gradient in NDVI; green-up began earlier in the south than in the northern portions of the study area. Individuals likely coordinate timing of spring migration in concert with slowly advancing plant phenology and displayed slower rates of movement with higher sinuosity during spring migration than during the fall when plant phenology was no longer a factor [23–24,27]. Large tracts of native grasslands have been altered through tillage within our study area [71]. The fact that grasslands are consistently selected across scales and the importance of greenness during spring migration, confirms the need to conserve the remaining grasslands as both seasonal and migratory habitat for pronghorn within the Northern Sagebrush Steppe [4,30,71].

Environmental conditions can deteriorate quickly across the Northern Sagebrush Steppe during fall with sudden fluctuations in temperature and precipitation. In response, fall migration in pronghorn is more rapid and directional (less sinuous) than spring migration [24]. Pronghorn across the study area typically move south during fall to frequently used winter ranges to locate favorable conditions (i.e., warmer temperatures, less snow cover) and gain access to large sagebrush stands, which provide greater nutrition than senesced grasses and forbs. We found that neither forage productivity (measured using NDVI) or snow cover were significant factors influencing selection though both have been suggested to initiate fall migration [68,72,73]. While we did not demonstrate a vegetation senescence factor in driving fall migration, [35] did report NDVI as a significant factor in the selection of winter ranges by Northern Sagebrush Steppe migratory pronghorn. Annual snow cover variation was observed across the study area, most notably during the fall period (October-November), which may be a prominent factor to observe yearly differences in migratory start and end dates, as well as overall distances moved during fall migrations [24]. We assessed fluctuating snow cover conditions across the Northern Sagebrush Steppe using satellite imagery and found snow cover did not significantly influence fall migration pathway selection. This contrasts with other studies that found snow cover did affect pronghorn movements [22,27,72]. The MODIS imagery resolution for snow occurrence used in our analyses (500 m^2^/pixel) may have been too coarse to truly model its effects on pronghorn migration. Further, MODIS imagery only provided information about the presence or absence of snow cover and did not provide additional information regarding depth or condition of snow cover, i.e. attributes shown previously to affect pronghorn movement behavior. Interestingly, Northern Sagebrush Steppe migratory pronghorn did not select against snow cover while on winter range [35]. More informative products are needed to accurately assess the effects of snow on pronghorn migration and seasonal habitat selection. Nonetheless, it appears that pronghorn in our study area do not initiate fall migration in response to senescing vegetation or snow cover but instead select for linear, un-fragmented fall migratory routes to winter ranges, through native grassland cover or exposed (barren ground) areas.

Interestingly, pronghorn selected against following large drainages during spring migration, despite higher forage productivity present there. However, during fall migration pronghorn selected to follow these major drainages as individuals and/or groups are inclined to migrate rapidly in an undistracted fashion to arrive efficiently at winter destinations [23,72]. The pronghorn’s eyesight is highly adapted to detecting movement over long distances and they have the ability to respond to perceived threats by rapidly accelerating to running speeds that exceed any existing predator in the prairie landscape [74]. Yet pronghorn have poor ability to remove snow from forage by pawing and have difficulty moving through deep snow owing to a high foot-loading index [75]. We suggest that the taller vegetation, rough terrain, and remnant snow present in typical Northern Sagebrush Steppe coulees and river valleys, are avoided by pronghorn when possible during spring, to reduce the risk of being ambushed by predators. While in the fall, pronghorn paralleled major drainages, following exposed ridgelines and along open south-facing slopes, which are typically snow-free owing to insolation and wind action.

The use of multi-scale maps in movement modelling shows promise in prediction of spatiotemporal resource selection that allows managers to account for variations in selection across scales, which are to be expected for most species. Other studies have found improved predictions when integrating seasonal range habitat predictions across scales [10,17]. However, it is conceivable that animals perceive spatial scales differently during migration events. We would like to see additional research concerning the interplay of migration learning, fidelity to migratory routes and seasonal ranges, and behavioral responses to spatial heterogeneity at multiple scales to fully understand how predication of migratory habitat across scales can best be achieved.

## Conclusion and management implications

We found that multi-scale selection for migrating pronghorn could be described and predicted with integrated map outputs for use across the United States—Canada border. The Northern Sagebrush Steppe transboundary region is the northern terminus of the Great Plains ecosystem, encompassing the northern extent of many species’ ranges which span across this international border. It is apparent that for conservation, efforts must be made to address wildlife connectivity to maintain resilient and well-functioning ecosystems [76]. In order to maintain connectivity for pronghorn across the Northern Sagebrush Steppe, we provide three general recommendations: First, protect and manage native grasslands which pronghorn depend on as optimal migration habitat. Second, facilitate movement across highways and high-density road areas to allow pronghorn access to quality forage and escape deteriorating conditions. Finally, continue communication, data-sharing, and management between jurisdictions as pronghorn and other wildlife do not recognize human-created borders. The mapping of ungulate migration habitat is a topic of high conservation relevance. With this in mind, we conclude that in general, multi-scale modeling of ungulate migration routes facilitates spatially-explicit depictions of migration-relevant habitats for prioritization in landscape planning. Moreover, scale-integrated step selection function map outputs defined and expressed here provide a critical step in guiding management and conservation priorities across the Northern Sagebrush Steppe and, plausibly, other broad regions to address continued declines in global ungulate migrations where telemetry-based sampling is unavailable or incomplete.

## Supporting information

S1 Fig **Predicted values (lines) of the relative probability of use from second-order models for pronghorn migratory neighborhoods during spring (red) and fall (blue).** Lines are only predicted for covariates when included within the final model for each season. Also plotted are histograms of the proportionate distribution of available values across the range of each covariate.
(DOCX)

S2 Fig **Predicted values (lines) of the relative probability of use from third-order models for pronghorn migratory pathways during spring (red) and fall (blue).** Lines are only predicted for covariates when included within the final model for each season. Also plotted are histograms of the proportionate distribution of available values across the range of each covariate.
(DOCX)
