# Supplementary material for: Multi-scale habitat assessment of pronghorn migration routes
Source: PLoS One. 2020 Dec 4;15(12):e0241042. doi: 10.1371/journal.pone.0241042 (PMC7717543; doi:10.1371/journal.pone.0241042)

S2 Fig. Predicted values (lines) of the relative probability of use from third-order models for pronghorn migratory pathways during spring (red) and fall (blue). Lines are only predicted for covariates when included within the final model for each season. Also plotted are histograms of the proportionate distribution of available values across the range of each covariate.


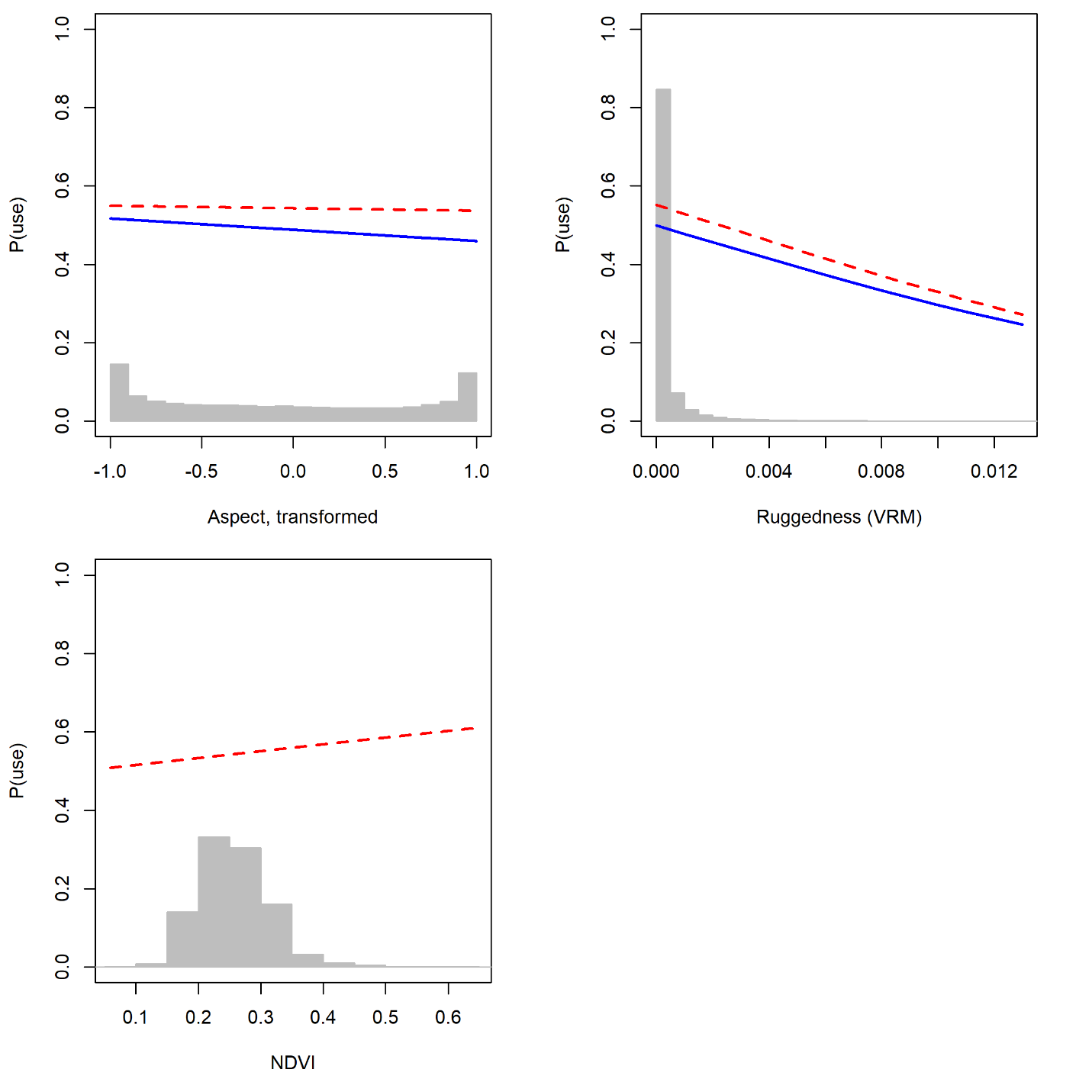

Supplement: S2 Fig — Predicted values (lines) of the relative probability of use from third-order models for pronghorn migratory pathways during spring (red) and fall (blue). Lines are only predicted for covariates when included within the final model for each season. Also plotted are histograms of the proportionate distribution of available values across the range of each covariate. (DOCX) [file pone.0241042.s002.docx]
